# Supplementary figures and images for: Generation and validation of versatile inducible CRISPRi embryonic stem cell and mouse model
Source: PLoS Biol. 2020 Nov 30;18(11):e3000749. doi: 10.1371/journal.pbio.3000749 (PMC7728392; doi:10.1371/journal.pbio.3000749)

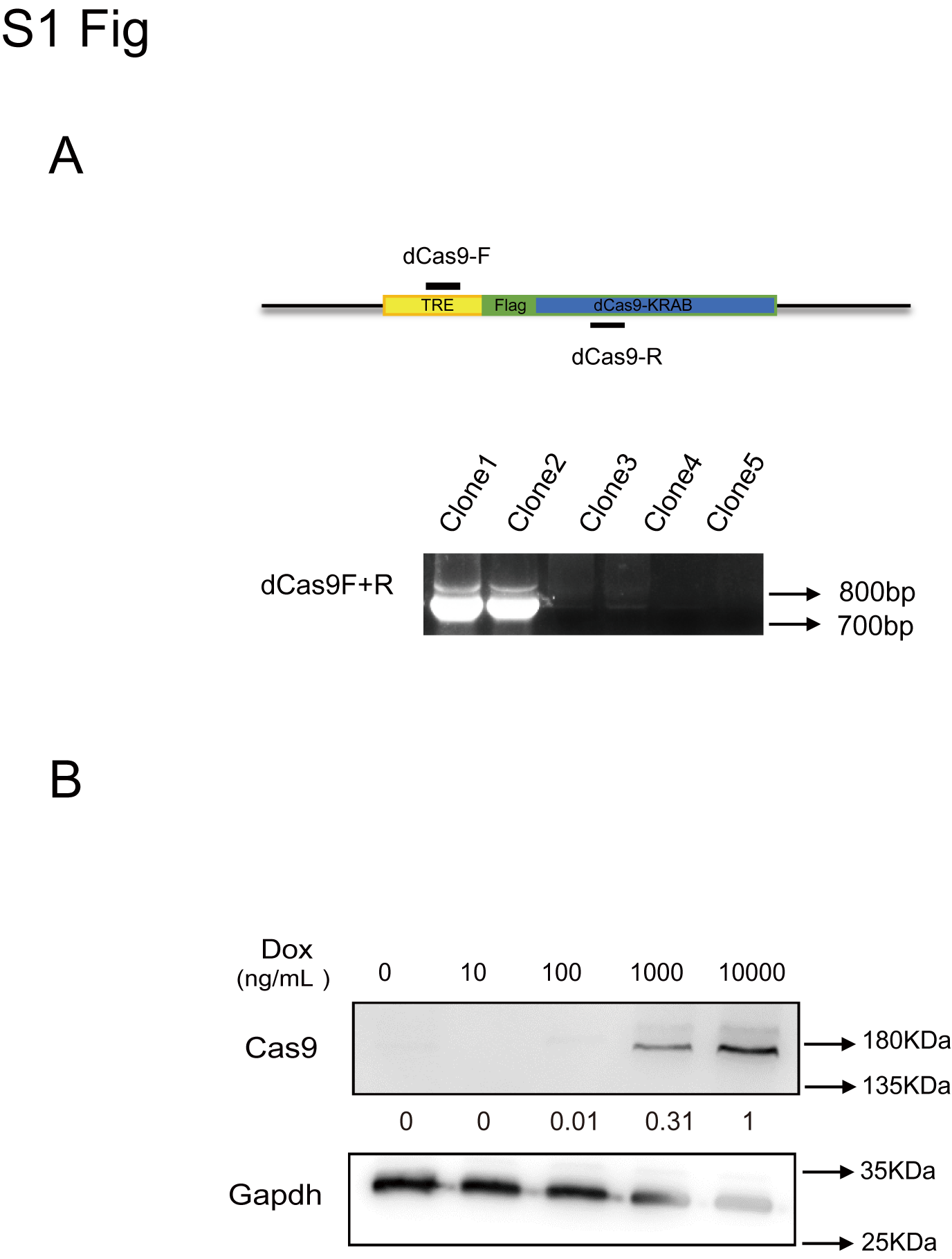

Supplement: S1 Fig — (A) Genotyping PCR analysis of proper integration of dCas9-KRAB at the designed locus. (B) Western blot analysis showing the inducible expression of FLAG-dCas9-KRAB protein by different concentration of Dox. Gapdh served as a loading control. A relative gray value quantification of dCas9-KRAB protein levels is below each lane of the band. dCas9, deactivated Cas9; Dox, doxycycline; ESC, embryonic stem cell; KRAB, Krüppel-associated box; PCR, polymerase chain reaction. (TIF) [file pbio.3000749.s001.tif]

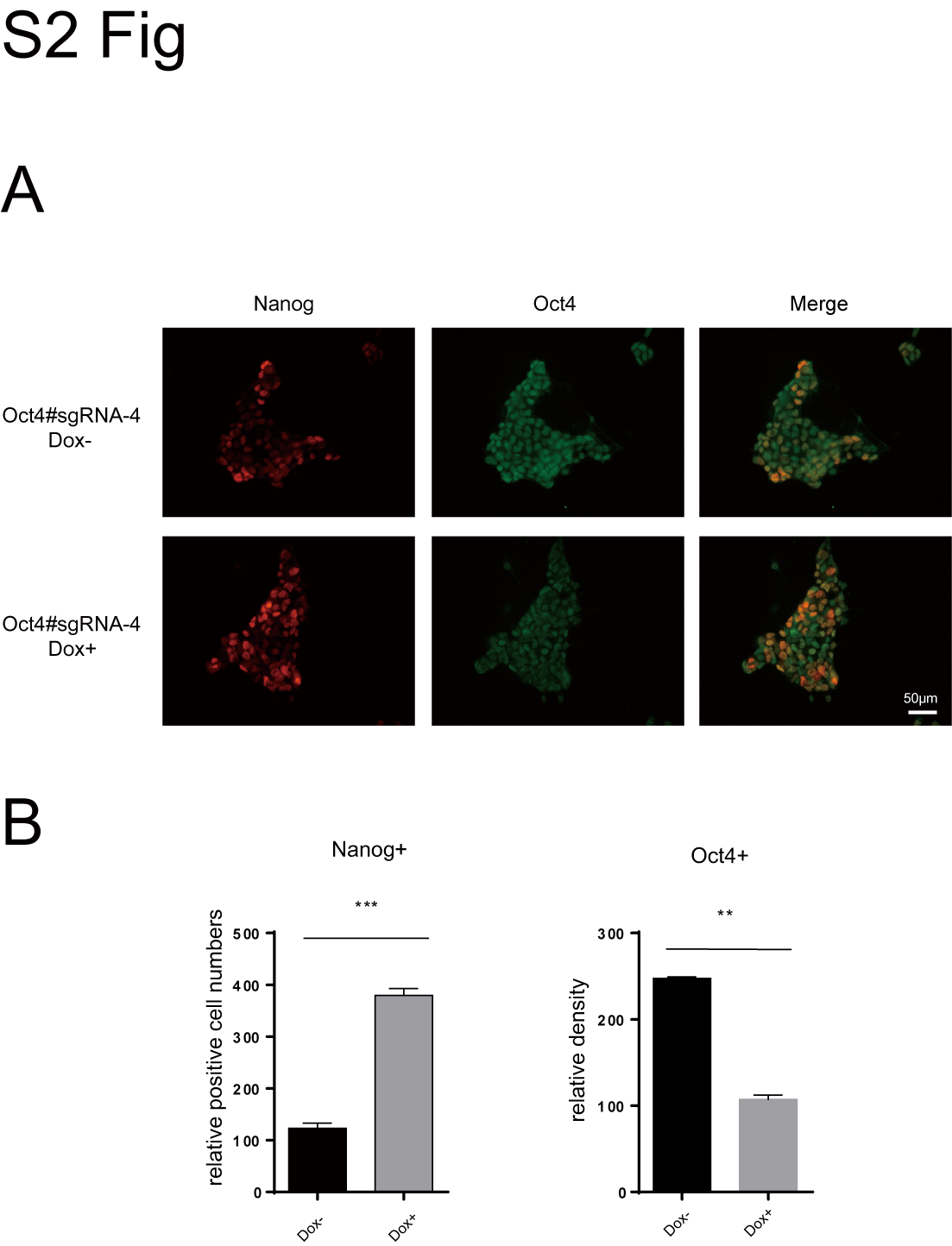

Supplement: S2 Fig — (A) Oct4 expression levels are down-regulated around 50% accompanied with higher rate of Nanog-positive cells. IF staining of Oct4 and Nanog in SL-cultured iKRAB cells containing Oct4#sgRNA-4 treated with or without Dox. The scale bar represents 50 μm. Right panel: (B) The relative density of Oct4 and the relative Nanog-positive cell numbers are compared in designated group. Data are represented as the mean ± SD of replicates (n = 3). (**p < 0.01, ***p < 0.001; 2-tailed unpaired t test). The numerical values used to generate graphs in panel B are available in S1 Data. CRISPRi, CRISPR interference; Dox, doxycycline; IF, immunofluorescence; SD, standard deviation. (TIF) [file pbio.3000749.s002.tif]

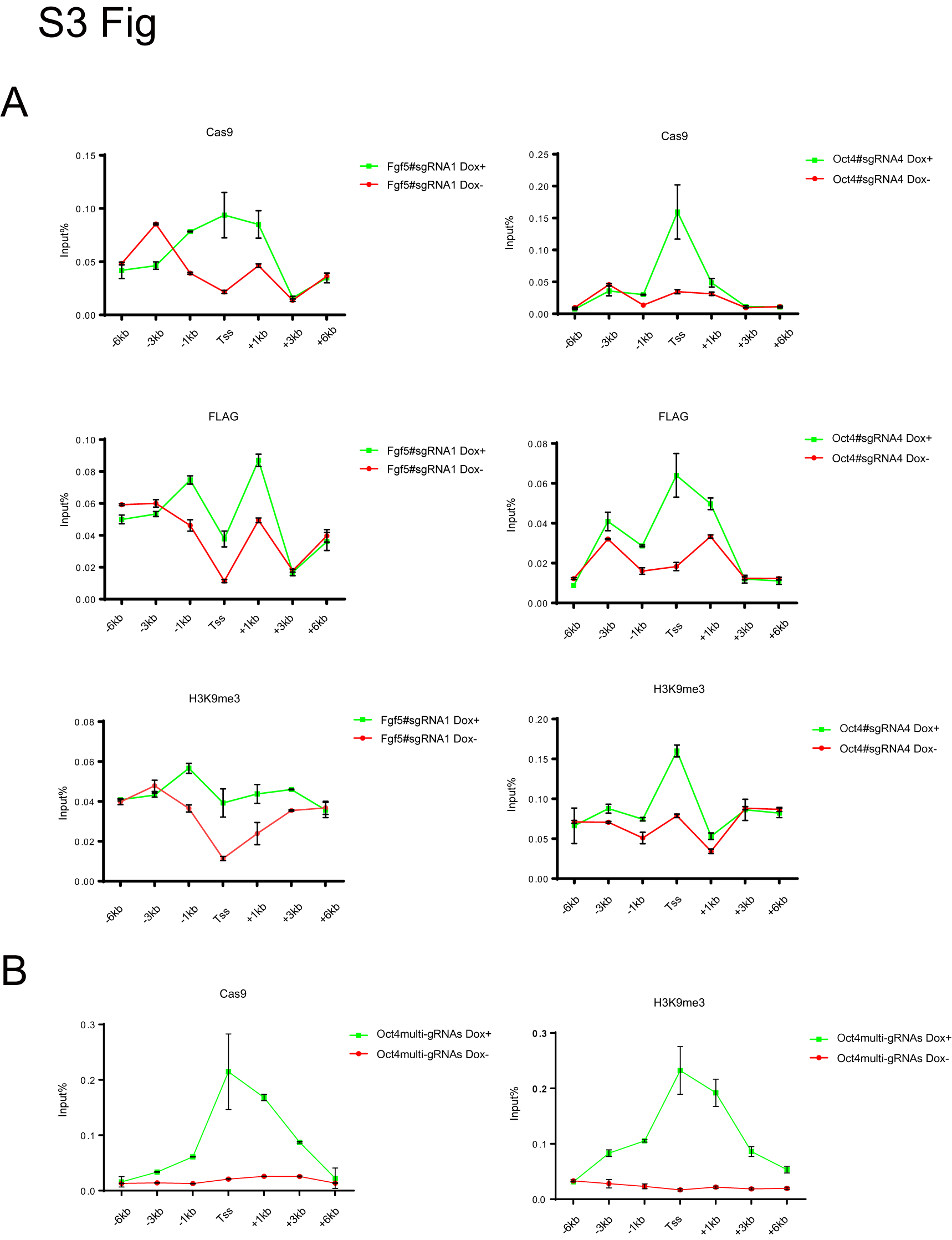

Supplement: S3 Fig — (A) ChIP-qPCR analysis of dCas9-KRAB guided by sgRNAs targeting around the TSS of Oct4 and Fgf5 with Cas9, FLAG, and H3K9me3 antibodies respectively. (B) ChIP-qPCR analysis of dCas9-KRAB guided by multi-gRNAs targeting around the TSS of Oct4 with Cas9 and H3K9me3 antibodies, respectively. Data are represented as the mean ± SD of replicates (n = 3). The numerical values are available in S1 Data. ChIP, chromatin immunoprecipitation; dCas9, deactivated Cas9; qPCR, quantitative polymerase chain reaction; SD, standard deviation; sgRNA, single-guide RNA; TSS, transcription start site. (TIF) [file pbio.3000749.s003.tif]

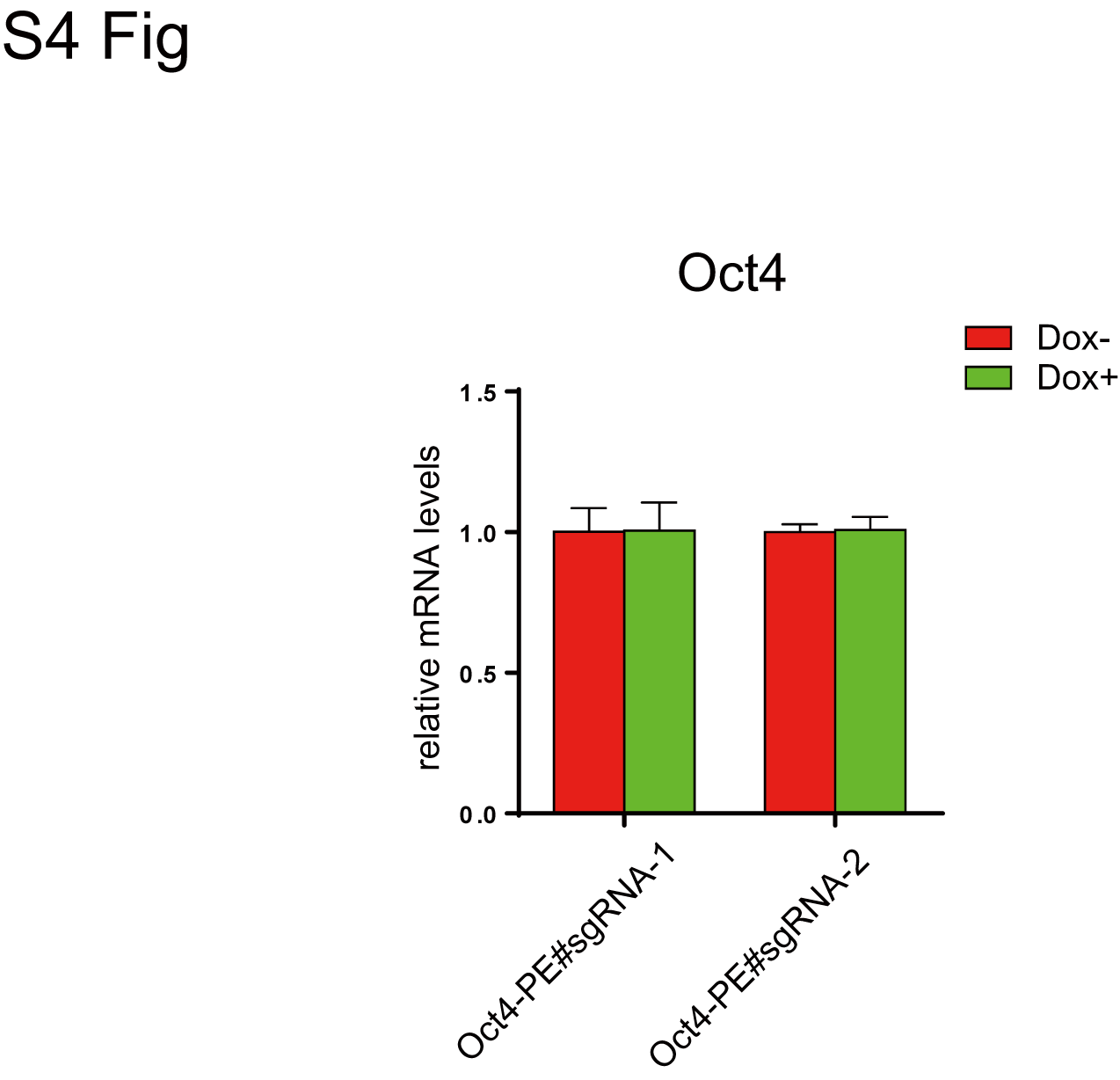

Supplement: S4 Fig — RT-qPCR analysis of Oct4 expression in stable iKRAB ESCs (2i condition) containing sgRNA against Oct4-PE. Data are represented as the mean ± SD of replicates (n = 3). The numerical values are available in S1 Data. CRISPRi, CRISPR interference; ESC, embryonic stem cell; PE, proximal enhancer; RT-qPCR, reverse transcription PCR; SD, standard deviation; sgRNA, single-guide RNA. (TIF) [file pbio.3000749.s004.tif]

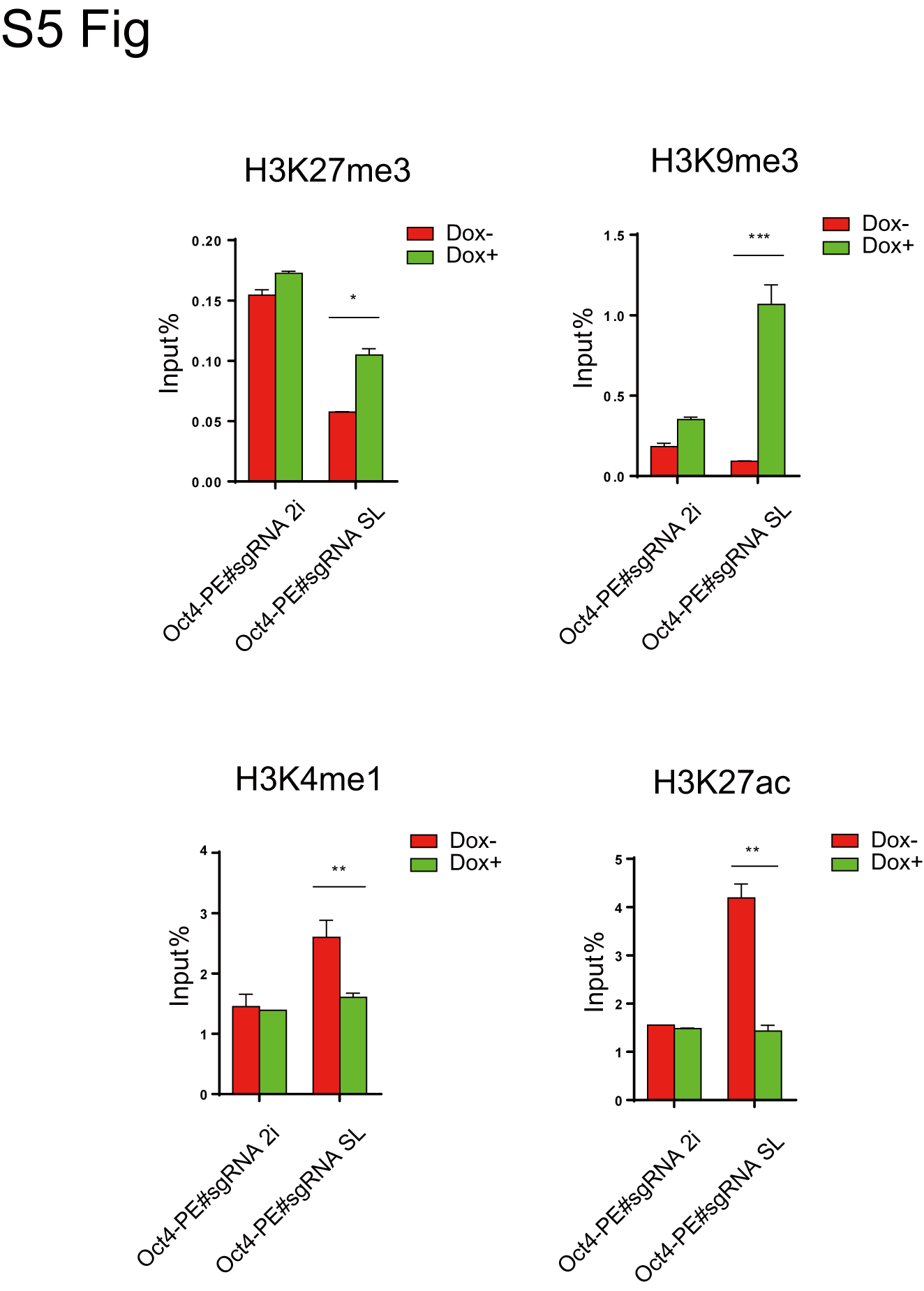

Supplement: S5 Fig — ChIP-qPCR analysis of epigenomic alterations at PE of Oct4 with or without Dox treatment during switch from 2i to SL conditions. Data are represented as the mean ± SD of replicates (n = 3) (***p < 0.001, **p < 0.01, *p < 0.05; 2-tailed unpaired t test). The numerical values are available in S1 Data. ChIP, chromatin immunoprecipitation; CRISPRi, CRISPR interference; Dox, doxycycline; PE, proximal enhancer; qPCR, quantitative polymerase chain reaction; SD, standard deviation. (TIF) [file pbio.3000749.s005.tif]

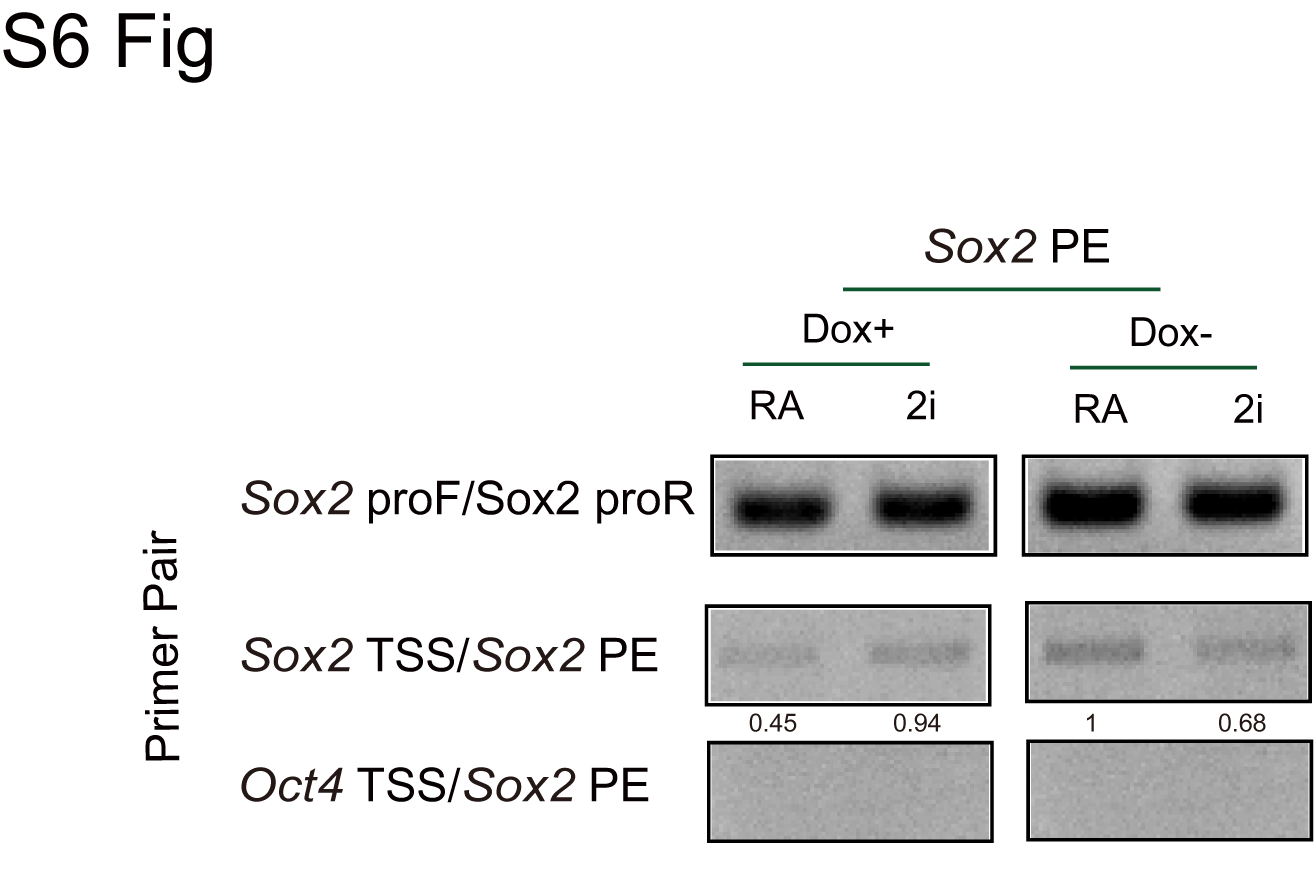

Supplement: S6 Fig — 3C-PCR analysis of Sox2 PE in designed groups. The primers tested the interaction between Oct4-TSS and Sox2-PE served as a negative control. A relative gray value quantification of PCR products is below each lane of the band. ChIP, chromatin immunoprecipitation; PCR, polymerase chain reaction; PE, proximal enhancer; TSS, transcription start site. (TIF) [file pbio.3000749.s006.tif]

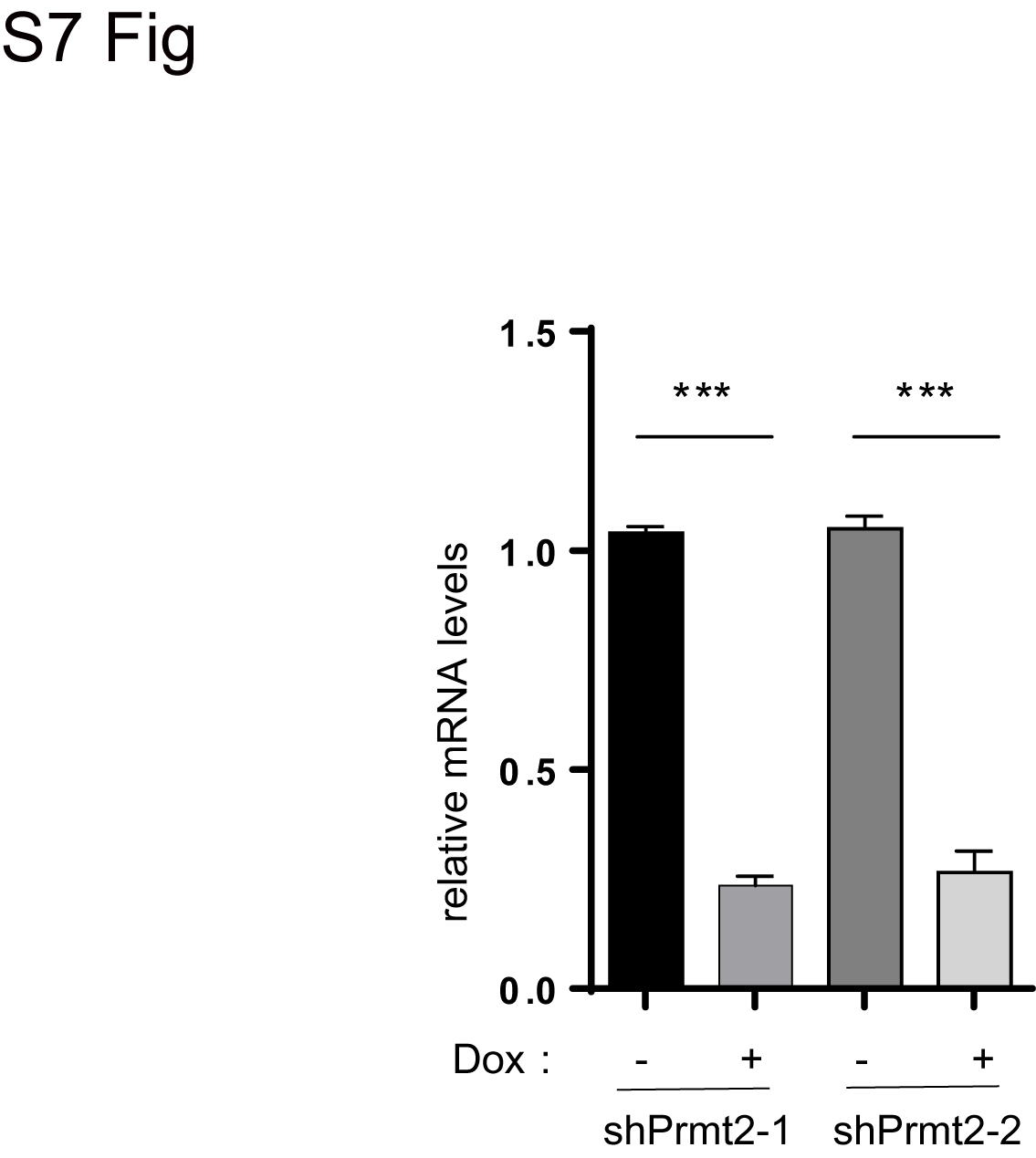

Supplement: S7 Fig — RT-qPCR analysis of Prmt2 expression in the shPrmt2-transduced cells (shPrmt2-1 and -2) with or without Dox induction. Data are represented as the mean ± SD of replicates (n = 3) (***p < 0.001; 2-tailed unpaired t test). The numerical values are available in S1 Data. Dox, doxycycline; RT-qPCR, reverse transcription PCR; SD, standard deviation. (TIF) [file pbio.3000749.s007.tif]
