# Supplementary material for: Generation and validation of versatile inducible CRISPRi embryonic stem cell and mouse model
Source: PLoS Biol. 2020 Nov 30;18(11):e3000749. doi: 10.1371/journal.pbio.3000749 (PMC7728392; doi:10.1371/journal.pbio.3000749)
Supplement: S2 Table — sgRNA, single-guide RNA. (DOCX) [file pbio.3000749.s009.docx]

**S2 Table. SgRNA sequences**

| name | sgRNA sequence |
| --- | --- |
| Oct4#sgRNA-1 | TGAGGCGAGCGCTATCTGCC |
| Oct4#sgRNA-2 | CAATCCCACCCTCTAGCCTT |
| Oct4#sgRNA-3 | CCCCAATCCCACCCTCTAGC |
| Oct4#sgRNA-4 | AAGGTGGGCACCCCGAGCCG |
| Oct4#sgRNA-5 | TCCACCAGGCCCCCGGCTCG |
| Oct4#sgRNA-6 | GCTGGGTGGATCCTCGAACC |
| Bap1#sgRNA-1 | TTAGCGGTCCTCGTTTTGGC |
| Bap1#sgRNA-2 | GCCCCCGGGTTAGGCTCGTC |
| Bap1#sgRNA-3 | GCGCGGGCGAGTGACGCGGC |
| Bap1#sgRNA-4 | TGCTAAACCCGCGCCGCCCC |
| Fgf5#sgRNA-1 | CCCCGTACCGGCCGTGAGTA |
| Fgf5#sgRNA-2 | GCGCCGAGATCCGCTCGGGT |
| Mll1#sgRNA-1 | CTTCACTTACGGGGCGAACA |
| Mll1#sgRNA-2 | AAGGCCGGGACGCGTTGCCG |
| Oct4PE#sgRNA-1 | ACTGGTTTGTGAGGTGTCCG |
| Oct4PE#sgRNA-2 | GCTGAGTGGGCTGTAAGGAC |
| Sox2PE#sgRNA | ACACTCGGCGGGGTGCACGT |
| Runx2#sgRNA | CTTCAGCGCAGTGACACCGT |
| Tfam#sgRNA-1 | GGGGGTAAGCCGCCGCTCAG |
| Tfam#sgRNA-2 | CGTCTATCAGGTAGGCCGCG |
| Tfam#sgRNA-3 | ATTTCTAATTGCTCGGTGCC |
| Oct4 multi-gRNA-1 | TGAGGCGAGCGCTATCTGCC |
| Oct4 multi-gRNA-2 | CCCCAATCCCACCCTCTAGC |
| Oct4 multi-gRNA-3 | CTAAGTTGCAGCGTGTGAAC |
| Nanog multi-gRNA-1 | TCCCCCCAAACCTGAGTCTT |
| Nanog multi-gRNA-2 | CGGTCACGGTTCTAATGAAG |
| Nanog multi-gRNA-3 | ACAATACTCAGTTCAATGGC |
| mPrmt2KD-1 | GCCAAAGTCGAATCATATCTT |
| mPrmt2KD-2 | GCTGTGTATATAGGTGTTCAT |
