# Supplementary material for: Generation and validation of versatile inducible CRISPRi embryonic stem cell and mouse model
Source: PLoS Biol. 2020 Nov 30;18(11):e3000749. doi: 10.1371/journal.pbio.3000749 (PMC7728392; doi:10.1371/journal.pbio.3000749)
Supplement: S3 Table — RT-qPCR, reverse transcription PCR. (DOCX) [file pbio.3000749.s010.docx]

**S3 Table. RT-qPCR primers**

| name | Primer sequences |
| --- | --- |
| Oct4-F | AGATCACTCACATCGCCAATCA |
| Oct4-R | CGCCGGTTACAGAACCATACTC |
| Runx2-F | AGTTTGCAAGCAGACCTTTGG |
| Runx2-R | CTTTCAGGGCTGCTTCGGAT |
| Bap1-F | CAACCTGATGGCAGTGGTGC |
| Bap1-R | CTTGTTGCTGGCTGACTTGGA |
| Fgf5-F | AAACTCCATGCAAGTGCCAAAT |
| Fgf5-R | TCTCGGCCTGTCTTTTCAGTTC |
| Mll1-F | CCAGCTACTCGCCTACACAG |
| Mll1-R | AGAGCCAATGCTCCGAAGAC |
| Prmt2-F | CTCGGACCACCAAGTACCAC |
| Prmt2-R | AAAGCCATTCTGCAGGACCA |
