# Supplementary material for: Generation and validation of versatile inducible CRISPRi embryonic stem cell and mouse model
Source: PLoS Biol. 2020 Nov 30;18(11):e3000749. doi: 10.1371/journal.pbio.3000749 (PMC7728392; doi:10.1371/journal.pbio.3000749)
Supplement: S4 Table — ChIP, chromatin immunoprecipitation; RT-qPCR, reverse transcription PCR. (DOCX) [file pbio.3000749.s011.docx]

**S4 Table. ChIP-qPCR primers**

| name | Primer sequences |
| --- | --- |
| Fgf5TSS-R | CACATGTGTCCCAGCCAACT |
| Oct4PE-F | ACTGGTTTGTGAGGTGTCCG |
| Oct4PE-R | GACAACCCTTAGGACGGGAC |
| Fgf5--6k-F | AGGCACAAATTATAGCAACAT |
| Fgf5--6k-R | TATACTAGCTGGAATTGAATT |
| Fgf5--3k-F | TGAGGAATGAATGACACCAT |
| Fgf5--3k-R | CTTTCTAAAAGGCATTCACCC |
| Fgf5--1k-F | CAGGTGACTGGCTATCAGCT |
| Fgf5--1k-R | TGTTAAGACATGACTTTAT |
| Fgf5-+1k-F | GCTCCTCTTGTCTTCCTGGTGG |
| Fgf5-+1k-R | CCATCCCACCTGTGCTTGAAT |
| Fgf5-+3k-F | AGCATCTACTTGCAACTGATT |
| Fgf5-+3k-R | TCATACTGAGGGTTATTTCC |
| Fgf5-+6k-F | TAGTATATGAGTGAATGCAC |
| Fgf5-+6k-R | ATGTAAACACCACATATGTG |
| Oct4--6k-F | AAGGTGGGCACCCCGAGCCG |
| Oct4--6k-R | CAGTACTAGTACGTGGATGTCT |
| Oct4--3k-F | AGCCTGGATGCTAACACGA |
| Oct4--3k-R | CAGCCTCAGCAGGAGACCTT |
| Oct4--1k-F | TCCTGGCCCATTCAAGGGTT |
| Oct4--1k-R | AGAAGAAATATCTGACTTCAG |
| Oct4-TSS-F | TGTGGGCGTCAGAGGTGGTC |
| Oct4-TSS-R | CTGTACTAGAGTGCGACAGA |
| Oct4-+1k-F | TAGCTGAATTTTGTTTTCCTTC |
| Oct4-+1k-R | TAGGATGGCGAATATGCAAT |
| Oct4-+3k-F | ACTGAGGCAGGAGAGCTTCCT |
| Oct4-+3k-R | AATTCCCAGCAACCACAAGGT |
| Oct4-+6k-F | AGAATAGGTCAAAGGCTTTAG |
| Oct4-+6k-R | TCAGGGACAGCCTGGCCTACA |
