# Supplementary material for: Generation and validation of versatile inducible CRISPRi embryonic stem cell and mouse model
Source: PLoS Biol. 2020 Nov 30;18(11):e3000749. doi: 10.1371/journal.pbio.3000749 (PMC7728392; doi:10.1371/journal.pbio.3000749)
Supplement: S5 Table — PCR, polymerase chain reaction. (DOCX) [file pbio.3000749.s012.docx]

**S5 Table. 3C-PCR primers**

| name | Primer sequences |
| --- | --- |
| Sox2Pro-F | AGTATCAGGAGTTGTCAAGGCA |
| Sox2Pro-R | CCGCCGCGATTGTTGTGATTA |
| Sox2PE | TCGAGGTTCTTCTGCAAAGGC |
| Oct4TSS | GGACACCTGGCTTCAGACTT |
| Sox2TSS | CATCCCAATTGCACTTCGCC |
