# Supplementary material for: Generation and validation of versatile inducible CRISPRi embryonic stem cell and mouse model
Source: PLoS Biol. 2020 Nov 30;18(11):e3000749. doi: 10.1371/journal.pbio.3000749 (PMC7728392; doi:10.1371/journal.pbio.3000749)
Supplement: S6 Table — NGS, next generation sequencing; PCR, polymerase chain reaction; sgRNA, single-guide RNA. (DOCX) [file pbio.3000749.s013.docx]

**S6 Table. SgRNA and NGS library construction and genotyping-PCR primers**

| name | Primer sequences |
| --- | --- |
| oligoAmp-F | TTGTGGAAGGACGAAACACCGATCGCGAAGACAACACCG |
| oligoAmp-R | TTGCTATTTCTAGCTCTAAATATGCAGAAGACTGTAAAAC |
| NGS-F | TTGTGGAAGGACGAAACACC |
| NGS-R | ACTTGCTATTTCTAGCTCTAAAT |
| rtTA genotyping-1 | AAAGTCGCTCTGAGTTGTTAT |
| rtTA genotyping-2 | GCGAAGAGTTTGTCCTCAACC |
| rtTA genotyping-3 | GGAGCGGGAGAAATGGATATG |
