# Supplementary material for: Generation and validation of versatile inducible CRISPRi embryonic stem cell and mouse model
Source: PLoS Biol. 2020 Nov 30;18(11):e3000749. doi: 10.1371/journal.pbio.3000749 (PMC7728392; doi:10.1371/journal.pbio.3000749)
Supplement: S7 Table — (DOCX) [file pbio.3000749.s014.docx]

**S7 Table. Antibodies in this study**

| name | Company | Cat. number | Applications |
| --- | --- | --- | --- |
| H3K27me3 | Cell Signaling Technology | 9733s | ChIP |
| H3K9me3 | Cell Signaling Technology | 13969s | ChIP |
| H3K4me3 | Cell Signaling Technology | 9751s | ChIP |
| H3K4me1 | Cell Signaling Technology | 5326P | ChIP |
| H3K27ac | abcam | ab4729 | ChIP |
| Cas9(7A9-3A3) mouse mAb | Cell Signaling Technology | 14697s | WB, IF, ChIP |
| Anti-Oct4 antibody [GT486] | abcam | ab184665 | IF |
| Anti-Nanog antibody | BETHYL | A300-397A | IF |
| Anti-Map2 antibody | abcam | ab183830 | IF |
| Anti-Flag antibody | sigma | F7425 | WB, IF, ChIP |
| Anti-Laminin antibody | abcam | ab11575 | IF |
| Anti-GFP-tag antibody | ProteinTech | 50430-2-AP | IF |
