# Supplementary material for: Generation and validation of versatile inducible CRISPRi embryonic stem cell and mouse model
Source: PLoS Biol. 2020 Nov 30;18(11):e3000749. doi: 10.1371/journal.pbio.3000749 (PMC7728392; doi:10.1371/journal.pbio.3000749)
Supplement: Attachment — Submitted filename: response to Reviewers.docx [file pbio.3000749.s016.docx]

Dear Editors,

We thank the four reviewers and editors for their constructive comments about the manuscript. And we appreciate the time and details provided by each reviewer and have incorporated the suggested changes into the new version of the manuscript to the best of our ability. The revised manuscript has certainly benefited from the reviewers' insightful suggestions. We hope that you will be able to accept the revised version for publication in PLoS Biology.

**Point-to-point responses to the reviewers’ comments**

Reviewer #1: Overall, the authors present a resource that will be useful for the scientific community for basic studies as well as disease modeling in vitro and in vivo. However, much of their data is not quantified and there are logical gaps in some of their conclusions. These issues should be addressed prior to publication.

Major Comments:

From data presented in Fig 2 and Fig 5, it is difficult to conclude that CRISPRi by iKRAB is not efficient on actively transcribed genes.

-For example, the most efficient window for CRISPRi gRNA design is reportedly the 100nt window downstream of the TSS. For OCT4 and BAP1, while the authors show multiple gRNAs, some are outside this window and therefore less likely to be effective. The authors do identify a gRNA for OCT4 which shows 50% knockdown. Showing a range of knockdown efficiencies for different gRNAs is not convincing for their argument above.

We thank the reviewer for acknowledging the potential of our systems for a broad range of applications. Actually we have shared the cell and mouse models with several other labs for cooperation. According to the reviewer’s suggestion, we are more careful with the phrase in our conclusions. In our original version, we took a strong criterion for “efficient” CRISPRi, e.g. above 75% downregulation. We do agree with the reviewer’s suggestion about the gRNA design and we did find that the downregulation efficiency of the sgRNAs targeting TSS-downstream regions was higher than the others (Fig 2A, S2B Fig). Nevertheless, we have not observed any sgRNA achieving better than 75% downregulation of active genes. To avoid misunderstanding, in the revised manuscript we no more claim that the CRISPRi by iKRAB is “not efficient” for actively transcribed genes. Instead we acknowledge the mild or moderate but significant downregulation effects accordingly.

-If BAP1 triggers cell death as discussed by the authors, could the authors be selecting for cells with reduced BAP1 knockdown in Fig. S2B?

Yes, the Bap1-downregulated cells could survive but grow slower than control cells. We have got similar observations in some other cell models. A G2/M cell cycle arrest was observed in certain cell types we tested (unpublished). And this is consistent with BAP1’s roles of H2AK119 deubiquitylation in G2/M cell cycle phase.

-In Fig 5E, it appears Runx2 expression is already activated by day 5 of differentiation and gRNA activation significantly inhibits Runx2 expression, which is nice to see in the mouse model but seems to conflict with their conclusions that the CRISPRi system is inefficient for silencing active genes.

Yes, we did show that the Runx2 induction is significantly suppressed by sgRNAs and Dox treatment. But similar to the above explanation, the Runx2 downregulation efficiency is modest. Hence we are cautious to call it “silencing” and rephrase it in the revised text accordingly (Page 12).

-In the paper by Mandegar et al (Cell Stem Cell, 2016), they are able to largely eliminate NANOG expression in multiple iPSC clones using CRISPRi; how do the authors' reconcile these conclusions?

We did cite this paper in our manuscript and notice the discrepancies. A possible explanation is that the Conklin lab used human iPSC as models. As we know, human iPSCs are maintained in the primed pluripotent state in normal hESC culture condition, rather than the naïve pluripotent state. In another word, the active expression of NANOG is not favored in hESC culture condition and therefore more likely to be suppressed by iKRAB. We suppose that the downregulation efficiency will be lower if the cells are maintained in 5i+LIF or other possible naïve pluripotent state-maintaining conditions. Of course we cannot exclude other possibilities beyond our current understanding.

A significant amount of data used to draw conclusions is not quantified. A few examples are given below.

-Fig S2A (reportedly showing homogeneous downregulation of OCT4) is not at all convincing. These data should be quantified (e.g., Western blot analysis).

-Similarly, other immunostaining figures throughout the manuscript used to draw conclusions (e.g., Fig 2D, 3C, 3G, 4C) should be quantified.

-In Fig 4C, again the data needs to be quantitative in order to any draw conclusions and in this case, the data underlying their conclusions seems particularly weak. From the single images, it appears AMI-1+ alone (upper right) and A803467+ alone (bottom left) significantly alter cell morphology and/or viability. And it is difficult to tell what's going on in the AMI-1+ and A803467+ condition (bottom right).

We appreciate the reviewer’s critical comments on these data. As suggested, we quantified the density of bands in WB assays and a relative gray value is now shown in new Fig 1B, 6C and S1B Fig. And we quantified and compared the relative positive cell numbers in multiple captured images for the all the IF assays as shown in new Fig 3D and G, Fig 4D and I, 5D, and S2A Fig. Moreover, we quantified the strength of *Sox2* enhancer-promoter interaction as examined by the 3C-PCR assays in new S6 Fig.

As for the validation of the screen, we did realize that the inadequacy of our previous experiments. The overdose of AMI-1 led to toxicity in the old data. Here we lowered the concentration of AMI-1 to repeat the assays during our revision. And we quantified the viable cells in each group accordingly. As shown in new Fig 5E and F, the AMI-1 does not show obvious toxicity at concentration of 4 or 8 μM and its addition significantly prevents cell death at the presence of A803467, arguing for its neuroprotective roles. To make the data more convincing and prevent the non-specific effects of the PRMT inhibitor, we knocked down PRMT2 by inducible shRNAs to validate its specificity and activity in counteracting against the toxicity of A803467 (new Fig 5C and D).

-Fig 3G is insufficient to conclude differentiation defects. Are these fewer neurons? Less MAP2 per neuron?

To make the data more conclusive, we repeated the assay and did more careful analysis. For one thing, we compared the IF density of MAP2 in the designated groups of cells. As shown in new Fig 4H and I, majority of the differentiated cells generated from the sgRNA-transduced iKRAB ESCs hardly express any detectable MAP2 and statistically the MAP2-positive cell numbers are significantly lower than the control group. It indicates the inactivation of Sox2 during differentiation compromises the subsequent generation of neurons.

RT-qPCR analyses:

-Similar fold changes are met with different conclusions by the authors. For example, all of the comparisons in Fig. 3B are significant (**), but based on the text, we are to conclude that the 2i comparison is not relevant, but the SL and FA conditions are.

As suggested, we repeated the RT-qPCR analysis and the data is much improved. As shown by the new Fig 4B and S4 Fig, no significant differences of Oct4 expression in 2i condition is observed. And more significant differences in SL and FA conditions is represented. The previous difference of Oct4 expression levels in 2i condition was confusing and probably just technical bias.

-The authors conclude that the switch-on of Sox2 PE was blocked in Fig 3E but with essentially the same fold change and significance values, the authors conclude that OCT4 sgRNA#4 (Fig 2A) was of poor efficiency.

We sincerely appreciate the reviewer’s careful comparison of the CRISPRi effects in different assays. As pointed by the reviewer, the downregulation of Sox2 expression was indeed inadequate in old Fig 3E. We suppose that it is due to the inadequate activation of Sox2 at Day 6 of NPC differentiation. Hence we repeated the whole differentiation and Dox induction processes. And we harvested the cells at Day 8 of NPC differentiation for RT-qPCR analysis. As shown in new Fig 4F, the suppression of Sox2 expression is more obvious and significant than previous data from Day 6 samples.

-In line 186-187, the authors reference a slight upregulation of OCT4 expression as shown by RT-qPCR (Fig 3A and S4) but Fig 3A is a schematic and the RT-qPCR data in Fig S4 shows no change. Should this be Fig 3B? If Fig 3B, why does OCT4 go up significantly when targeted under 2i conditions?

-It would be helpful to include fold-change for different conditions, as the reader has to compare relative changes across different baselines in the current RT-qPCR graphs.

As explained above, the data from old Fig 3B was probably just technical bias and we repeated the analysis and the corrected data is now shown in new Fig 4B.

Minor comments

-Please be consistent with nomenclature (e.g., gRNA and sgRNA used interchangeably).

Actually we use different nomenclature to distinguish single gRNAs from multiple gRNAs. When we mention gRNA, it refers to either sgRNA or multi-gRNAs.

-The labeling of Fig 6C, D seems off (lines 289-293) as Fig 6D is never referenced in the text.

We thank the reviewer for the careful reading and have made the correction accordingly.

Reviewer #2: The submitted manuscript "Generation and Validation of Versatile Inducible CRISPRi Embryonic Stem Cell and Mouse Models" describes generation of mouse ES cells (iKRAB) and consequently mice containing an inducible dCas9-KRAB construct. The authors find that CRISPRi induction does not repress its target locus well at highly expressed genes referred to as "actively transcribed genes" (eg when targeting the Oct4 promoter). The authors leverage transition states of differentiating iKRAB ES cells and demonstrate that dCas9-KRAB is capable of maintaining a repressed state at promoters or block activation at gene proximal enhancers. Finally, mice expressing inducible dCas9-KRAB are created to test repression ex vivo and in vivo.

Although this manuscript holds a great premise, this reviewer does not feel that the current manuscript lives up to its claims. The authors have made interesting observations that appear to only apply to a subset of cell states. This manuscript is of interest to the field, but claims about versatility cannot be made. Especially, considering another recent study has developed enCRISPRi and enCRISPRa mice (PMID:31980609) and have demonstrated versatility of their system and shown functionality in several mouse tissues.

We thank the reviewer for acknowledging the promise of our systems. We do realize the recent rising of similar systems in the field, which is not surprising as epigenome editing is an important and interesting expansion of the field of genome editing. Nevertheless, our inducible system still holds its advantage. As demonstrated in our manuscript, we could control the CRISPRi effects by adjusting the dose and timing of Dox addition. No doubt this is critical for the spatiotemporal control of gene expression for developmental and pathophysiological studies *in vivo*, which is not feasible for the enCRISPRi and enCRISPRa mice as mentioned by the reviewer. For example, we can compare the differential tolerance to Tfam loss in muscles by adjusting the virus injection and/or Dox addition at different ages, or in mice taking differet diets, etc. Therefore we believe that our inducible CRISPRi systems still provide an advantageous model for much broader applications.

Major concerns:

CRISPRi is a well-established system that has been used for high-throughput screens in many studies. In this study, the authors conclude: "Therefore our data argue against the high CRISPRi efficiency by iKRAB on actively transcribed genes". Did the authors intend to refer to highly expressed genes or any gene that is expressed and hence actively transcribed? This claim needs to be better defined and strengthened by careful evaluation of the effect of iKRAB on genes with various expression levels. This is a necessity to evaluate the versatility of these cells.

The authors then identify very specific developmental stages to evaluate their CRISPRi ES cell line. In these particular situations of cell state transitions, the authors show convincingly that dCas-KRAB induction can maintain repression of inactive genes when targeting promoters or proximal enhancers. While these are interesting observations for iKRAB cells, they only apply to particular cell states and transitions and hence will have limited applications.

We thank the reviewer for acknowledging our observations interesting. We do realize that dCas9-KRAB is a broadly used CRISPRi system. The authors have been working on epigenetic regulations for a dozens of years. We believe that our observations fit well with the current understanding of chromatin biology. At active genes, the sustained activation signals disfavor the establishment and propagation of repressive chromatin states. And loss of activation signals usually precedes transcriptional repression and deposition of repressive chromatin marks. In another word, chromatin modifications or modifiers are usually not determining factors of transcription. Moreover, transcription regulation is a multi-step process including initiation, elongation and termination. Hence transcriptional levels can hardly be a precise criterion to predict CRISPRi efficiency. So we thank the reviewer for providing the possible solutions to strengthen our argument. But we do not think it possible or feasible to simply evaluate the possibly varied repressive effects on target genes with different expression levels.

Notably, modest CRISPRi effects by sgRNAs and highly efficient CRISPRi effects by multi-gRNAs can still be achieved in our system. Moreover, the pre-set dCas9-KRAB is sufficient to maintain the transcription repressive states. And considering of the inducibility of our systems, we can easily study the spatiotemporal activities of genes of interest by proper timing of Dox addition. Thus, while we understand such concerns of potential limits of applications that the reviewer raised, we must say however that they are really unnecessary. In addition to the applications that we demonstrated in our manuscript, our systems have actually been put into application by other labs. Here is one of the recent published paper using our system: Yu, J., et al. (2020). "Analysis of Local Chromatin States Reveals Gene Transcription Potential during Mouse Neural Progenitor Cell Differentiation." Cell Rep 32(4): 107953. And more cooperative work using the mouse models are ongoing.

The authors further demonstrate the use of iKRAB cells in a screen targeting promoters of chromatin regulators to identify genes involved in toxicity of sodium channel blockers. Here the authors chose gRNAs targeting gene promoters. This is an interesting experiment again taking advantage of transitioning cells and identifying 19 candidate genes. It is unclear if the experiments were carried with biological replicates as this would be important to demonstrate validity of these findings. More thorough experimental design and validation is needed to support these observations.

We sincerely appreciate the reviewer’s suggestions. Yes, the screening assays were performed with duplicates. For further validation, we tried inducible knockdown of Prmt2 during NPC differentiation. And interestingly Prmt2 depletion successfully prevents neuron death caused by the neurotoxic compound A803467 (new Fig 5C and D). These assays further illustrate attractive applications of our systems in loss-of-function screens.

When the iKRAB mouse model is used in combination with 3 gRNAs delivered to the muscle, significant reduction of TFAM is observed in vivo. All prior experiments are based on a single gRNA. Perhaps the iKRAB ES cells and mouse model would be more versatile when using multiple gRNAs. Could targeting of multiple guides to one target have a synergistic effect and more efficiently repress "actively transcribed genes"?

Yes, the multi-gRNAs targeting the TSS of Oct4 and Nanog did display stronger efficiency in the downregulation of these active genes (new Fig 2B).

Minor comments:

Authors need to clearly identify all plasmids obtained from other sources (Addgene #) and add reference for each of these plasmids.

We added the Addgene # accordingly in the revised manuscript.

Inducible protein expression was confirmed by Western. There should be some reference to which antibody and protocol was used. Which antibodies were used for ChIP? Please include company and catalog#

We clearly labelled all the above requested information in the updated S7 Table.

In all figures where applicable, please add sgRNA#s to corresponding position diagram. I assume they are graphed in the same order, but that would make it clearer.

We clearly labelled the sgRNA#s accoding to the suggestion in all the relevant figures.

Supplementary Figure S6. I did not see the difference between +/- DOX.

We quantified and compared the PCR band density by Image J. The relative gray values show that the enhancer-promoter interaction strength is increased in control group (Dox-: 0.68 to 1 during NPC differentiation) while decreased in Dox-treated group (0.94 to 0.45 during NPC differentiation). We believe that the Dox-induced CRISPRi at *Sox2*PE strongly suppressed the chromatin looping.

Reviewer #3: The goal of this study was to develop tools to suppress genes in murine ES cells and mice. The authors generated murine ES cells in which dCas9-KRAB was inserted into the Hprt locus under the control of a doxycycline (rtTA)-responsive promoter. They then demonstrate that introduction of sgRNAs targeting inactive, but not active, genes can maintain the target genes in an inactive state. They also demonstrate the utility of the ES cells by performing a high-throughput screen and by using them to create mice harboring the dCas9-KRAB cassette. Lastly, they use the novel mouse model to demonstrate suppression of a target gene in vivo.

Comments

1. A major limitation of the tools developed by the authors is the inability of the dCas9-KRAB transgene to silence active genes. Although the authors cite a study demonstrating a similar phenomenon, they do not acknowledge or discuss the numerous studies in which dCas9-KRAB effectively suppresses actively transcribing genes. For example, using an approach very similar to the authors, Conklin and co-workers created human iPSCs harboring a Dox-regulated dCas9-KRAB and showed that it effectively suppressed Nanog, which was robustly expressed prior to Dox addition (see Figure 2C in Cell Stem Cell 18:541, 2016). Indeed, the development of this technology by the Weissman and Qi labs for use in genome-wide screens depends on the ability of CRISPRi to suppress most target genes, irrespective of their initial transcriptional state. No explanation is provided for the lack of effectiveness in this particular model.

We did cite the studies that the reviewer mentioned (Ref [6, 8, 9, 19]) and realize the discrepancies. Actually we do agree that CRIPSRi by sgRNA-guided dCas9-KRAB could significantly suppress active gene transcription (new Fig2A, S2B Fig, Fig 3F). However we have to argue against the high efficiency. Epigenetic studies have demonstrated that the sustained activation signals disfavor the establishment and propagation of repressive chromatin states. In support of this concept, we found that the propagation of H3K9me3-positive heterochromatin-like chromatin state is counteracted by active transcription (new S3 Fig). Actually loss of activation signals usually precedes transcriptional repression and deposition of repressive chromatin marks. Hence the chromatin regulatory effects by epigenome editing techniques including our iKRAB system do NOT simply determine transcriptional levels. Therefore it is not really surprising to observe inadequate CRISPRi of active genes when using sgRNAs. This idea is further supported by the fact that higher downregulation efficiency is achieved when using multi-gRNAs (new Fig2B and Fig 7B).

Anyway we thank the reviewer for raising these concerns. Accordingly, we have done more careful analyses, made major changes of the manuscript’s structure, and provided more insightful discussion in the revised texts (Results and Discussion). Nevertheless, we do not think that this is a limitation of our systems. Our inducible systems provide eligibility to study the spatiotemporal activities of genes of interest by proper timing of Dox addition in a wide range of contexts. The LOF screen and in vivo LOF studies (new Fig 5 and Fig 7) in this manuscript already justify for its broad applicability. And other ongoing cooperative work between our lab and others further proves its attractiveness.

2. Another significant limitation is the lack of any demonstration of target gene specificity. While others have demonstrated relatively high specificity of the dCas9-KRAB system, whether such specificity exists in the ES cells and mice generated by the authors is unclear.

We thank the reviewer for this criticism. However target specificity is the commonest and biggest concern in the field of genome/epigenome editing. Our systems do not have any advantage or disadvantage in this perspective. As CRISPRi does not change DNA sequences, we cannot examine the specificity by genomic DNA sequencing. We did realize that RNA-seq analysis for differentially expressed genes was used in some studies to demonstrate CRISPRi specificity. But we do not believe that it is a proper way to justify the specificity. Accompanied with the CRISPRi of specific gene of interest, it is unavoidable to observe the downregulation of some other associated genes. Actually the hits identified from our LOF screens can be nicely validated by shRNAs and inhibitors (new Fig 5). These facts suggest that the concern of off-target possibilities is not necessary.

3. The statement on page 14 suggesting that in vivo use of dCas9-KRAB is challenging due to its large size is not supported by recent publications (Sci Reports 9:17312, 2019; Nature Neuroscience 21:447, 2018).

We were not saying that the *in vivo* delivery of dCas9-fusion genes is not possible but claiming that it is challenging. We did read the two references. As mentioned in the Methods section of these two papers, the authors used super high titer of virus for the *in vivo* injection. However it is not easy to achieve such a high titer of lentivirus in regular biology labs.

Reviewer #4: This manuscript presents data of using an inducible Crispr repression system to probe gene functions and epigenetic modulator-screening in mouse ES cells (iKRAB ESC), and in the mouse. The system is in general not a novel one since many similar studies have been published. They however present some interesting findings. For example, the system suppresses gene expression more effectively for the genes that are not initially expressed. For genes that are highly expressing in ESCs, targeting the promoters or TSS is not effective. This finding is in line with other studies where targeting suitable enhancers is much more effective than TSS or promoters for actively expressing genes. The CRISPRi screening in this study has identified toxicity resistance genes which may provide possible solutions for neuroprotection, which is interesting.

Since the rtTA is inserted into the Rosa26 locus and the TRE-dCas9-KRAB integrated at the Hprt, it is expected that these two transgenes are segregated into different germline transmission mice from chimeras. The authors need to state clearly how the mice carrying the two transgenes are generated for in vivo study.

We thank the reviewer for the positive comments of our systems and the relevant findings. And we appreciate that the reviewer did so careful analysis for the generation of our systems (Fig 1A). The strength to have rtTA and TRE-driven transgenes at different chromatin regions is to successfully avoid leakiness. And this is indeed an advantage of our system. The disadvantage is that we have to cross a big amount of mice each time to get enough homozygous mice with simultaneous transmission of the two transgenes. To make it clear to readers and potential users of our systems, we listed the primers for the genotyping rtTA (S6 Table) as well as for the transgene of TRE-dCas9-KRAB.

In the mouse experiment, the system is used to target the specific enhancer and the first exon of Mitochondrial transcription factor A (TFAM), whose depletion results in muscle atrophy. The data show that Tfam expression levels are significantly lower in GFP+ (AAV-gRNA-expressing) cells, which also have smaller diameter of muscle fibers, indicative of muscle atrophy. Have the authors performed any functional tests of these mice that have putative muscle atrophy?

According to the reviewer’s suggestion, we repeated the whole assay, including crossing to get enough mice, AAV injection in left or right hindlimbs and Dox induction. Then we tried the function tests of hindlimb grip strength after observing the muscle atrophy. As shown in new Fig 7E, the *in vivo* Tfam CRISPRi did lead to modest but significant weaker grip strength than the control group. These data provide strong evidence that our systems are qualified for *in vivo* functional studies.

Some details also appear missing. For example, the section describing Figure 6B-C is confusing, and apparently mis-cites figure panels. There is no citation of Figure 6D.

We are sorry for the carelessness and have made correction accordingly in the revised manuscript.
