# Supplementary material for: Generation and validation of versatile inducible CRISPRi embryonic stem cell and mouse model
Source: PLoS Biol. 2020 Nov 30;18(11):e3000749. doi: 10.1371/journal.pbio.3000749 (PMC7728392; doi:10.1371/journal.pbio.3000749)
Supplement: Attachment — Submitted filename: response to Reviewers 202010.docx [file pbio.3000749.s017.docx]

**Point-to-point responses to the reviewers’ comments**

Reviewer #1:

The authors present new data and analysis which overall increase the rigor of the manuscript and the resources they have developed will be useful to the scientific community. However, a few key issues remain which should be addressed prior to publication.

First, the author's conclusions about dCas9-KRAB exerting modest effects on active gene expression remain confusing and not entirely supported by the data.

-The manuscript contains a mix of statements like, "after introduction of specific gRNAs, the induced dCas9-KRAB efficiently maintains gene inactivation, though it exerts modest effects on active gene expression" (lines 38-39), "induced dCas9-KRAB displayed limited effects on active promoters or enhancers" (lines 337-338), and "multi-gRNAs targeting a wide-range of regions of the same gene mediate sufficient transcriptional repression" (lines 348-349). Overall, are the authors arguing that dCas9-KRAB has modest effects on active gene expression only when using sgRNAs but that this problem can be resolved by multiplexing? Or that dCas9-KRAB, regardless of single- or multi-gRNAs utilization, has limited effects on active gene expression? This is an important difference and it is not always clear from the text and figures which position the authors are taking. If the authors want to draw conclusions about single- versus multi-gRNAs, then using multi-gRNAs should indeed spread

the heterochromatin-like state in contrast to single-gRNAs (Fig S3). However, experiments like this were not done to directly test the hypothesis, if this is the position the authors want to argue. Their conclusions about single- versus multi-gRNAs remain limited; really just data shown for Oct4 for a direct comparison of single- versus multi- gRNA effects on a gene.

We thank the reviewer for acknowledging the improved quality of the manuscript and the potential applications of our systems by the scientific community. According to the reviewer’s suggestion, we clearly indicated that the CRISPRi effects by single guide RNAs are modest. We made this change in new Line 27. Moreover, we performed ChIP-qPCR analysis for the chromatin states after introduction of multi-gRNAs targeting the *Oct4* TSS. As shown in the new S3B Fig, H3K9me3 spreads more than 12 Kb around Oct4 TSS, in contrast to the narrow distribution targeted by sgRNAs. These data indicate that multi-gRNAs indeed lead to more widespread heterochromatin-like state than single-gRNAs and explain the difference of CRISPRi efficiency.

-In Fig 4B, there appears to be a rather dramatic effect with an Oct4 PE sgRNA in the SL conditions where the PE is active. Oct4 levels were referred to as "successfully repressed in the SL condition" in line 215 of the text. Was dox treatment given after the change from 2i to SL? Does this conflict with the conclusion of limited effects on active enhances?

We thank the reviewer for the careful analysis of the experimental conditions and reading of the manuscript. Actually the Dox treatment was initiated as shown by the scheme in Fig 4A. So this is consistent with our conclusion that the pre-set inactive *Oct4*-PE by induced dCas9-KRAB is resistant to the activation signal. To avoid misunderstanding, we made a minor change in new Fig 4 by clearly indicating the timing of Dox addition.

-The point raised previously about BAP1 potentially triggering cell death (and therefore selecting for cells with reduced BAP1 knockdown) means that it is not a great example to select to show limited CRISPRi efficiency. Can the authors show other examples of genes that would not have this confound?

We deleted this panel as these data do not have much effect on our conclusion.

There are a few concerns with regard to quantification of IF images.

-Quantification was presented as cell number / mm2, which requires uniform plating density of all cultures for accuracy. These types of analyses should anchor the signal relative to the number of DAPI positive cells.

As suggested by the reviewer, we re-quantified the IF data by calculating the relative positive cell numbers through normalization by the number of DAPI-positive cells. The changes were made in the new Fig 3DG, Fig 4DI, Fig 5D and S2B Fig.

-In some cases, it's not clear how quantification was performed. For example, in Fig S2, Oct4 looks present but of reduced intensity in some cells in panel A, but the authors appear to take a positive / not positive approach to quantification in panel B. How did they threshold and quantify images like this? Moreover, the conclusion that "Oct4 expression was homogeneously downregulated after Dox treatment" (lines 130-131) seems at odd with quantification showing a nearly three-fold reduction in the number of positive cells (panel B)?

As suggested by the reviewer, we turned to compare the Oct4 fluorescence intensity instead of cell numbers as Oct4 expression is reduced in all the cells. The changes were made in the new S2B Fig.

Finally, I am confused by Figure 3F/G. The authors state in lines 193-195 that "upon being switched back to 2i medium, a subset of the Mll1 knockdown cells regained Oct4 and Nanog expression. In contrast, Oct4 and Nanog-positive cells could hardly be observed in the control group." In Figure 3F/G, doesn't Mll1 knockdown (+dox) in 2i shows no Oct4 or Nanog expression in contrast to the control or -dox condition?

We thank the reviewer for the careful reading and apology for the carelessness. We mis-labelled the Dox+/- in the opposite and they are now corrected in the new Fig 3FG.

Reviewer #2: The authors have addressed all concerns.

We thank the reviewer for all the comments and help.

Reviewer #3: The responses are adequate except for the response to comment 3. The authors claim that in vivo delivery of dCas9 is challenging but still do not adequately support this contention. They mistakenly claim that the references I cited use "super high titer of virus". The definition of "super high" is debatable. However, one of the publications I cited does not use virus at all but instead used a transgene via pronuclear injection, a procedure that is not really challenging at all. I maintain that the authors should modify this statement without providing evidence to support it.

As suggested by the reviewer, we deleted this statement which may sound too subjective in the updated manuscript.
